# Coexistence of Primary GEJ Adenocarcinoma and Pedunculated Gastric Gastrointestinal Stromal Tumor

**DOI:** 10.1155/2018/4378368

**Published:** 2018-06-11

**Authors:** Aroub Alkaaki, Basma Abdulhadi, Murad Aljiffry, Mohammed Nassif, Haneen Al-Maghrabi, Ashraf A. Maghrabi

**Affiliations:** ^1^Department of Surgery, Faculty of Medicine, King Abdulaziz University, Jeddah, Saudi Arabia; ^2^Department of Pathology, King Faisal Specialist Hospital and Research Center, Jeddah, Saudi Arabia

## Abstract

Gastrointestinal stromal tumors (GISTs) are the most common mesenchymal tumors of the digestive system, although they account for only 0.1–3% of all gastrointestinal (GI) malignancies. They can arise anywhere along the GI tract with gastric predominance. Concurrent occurrence of GIST and gastroesophageal junction (GEJ) neoplasm is rare. We report a 55-year-old gentleman presenting with a polyp at the GEJ and a synchronous, large, and pedunculated gastric mass at the greater curvature. Those were treated with a wedge resection of the gastric pedunculated mass with negative margins along with transgastric submucosal resection of the GEJ polyp. Pathological examination confirmed synchronous invasive GEJ adenocarcinoma and a high-grade gastric GIST.

## 1. Introduction

Gastrointestinal stromal tumors (GIST) are the most common mesenchymal tumors of the gastrointestinal (GI) tract predominantly involving the stomach [1, 2]. Concurrent occurrence of GIST and gastroesophageal junction neoplasms has rarely been reported in the literature [3]. We report a case of a 55-year-old gentleman with an incidental finding of a synchronous pedunculated gastric GIST and a polyp containing adenocarcinoma at the gastroesophageal junction.

## 2. Case Report

A 55-year-old gentleman, ex-smoker, presented to our hospital complaining of mild epigastric pain, regurgitation, and heartburn. On top of that, he has a long-standing history of gastroesophageal reflux disease (GERD), which was managed by proton pump inhibitors. His past medical history was significant for hypertension. He was previously diagnosed with a liver hemangioma based on abdominal ultrasound two years before the presentation. He had no relevant family history. Physical examination revealed mild epigastric tenderness with no palpable abdominal mass. Laboratory data showed no anemia but positive stool occult blood test. Tumor markers including AFP, CEA, and CA 19-9 were all within normal range. Upper GI endoscopy revealed mild esophagitis, Los Angles grade A along with Barrett's esophagus without dysplasia and a 1 cm polyp at the GEJ. A sample was sent for histopathology; the rest of the stomach and duodenum were normal. The patient did not have a previous endoscopy prior to this one.

**Figure 1:**
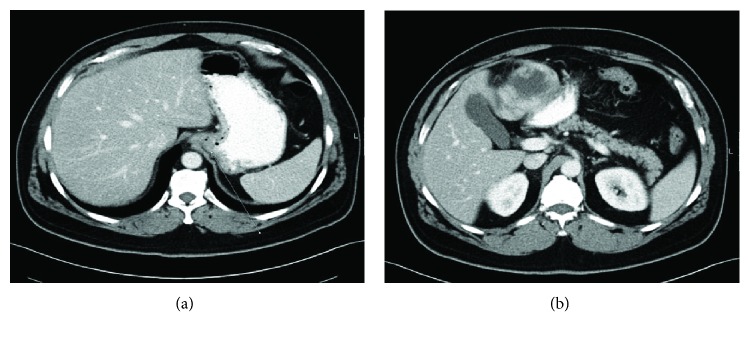
Infused CT of the abdomen. (a) Circumferential thickening of the lower esophagus. (b) A large mass with peripheral enhancement and central necrosis most likely representing gastric GIST.

Infused computed tomography (CT) of the abdomen and chest showed mild GEJ thickness with no evidence of mediastinal or celiac lymphadenopathy and no signs of metastasis. It also demonstrated a large heterogeneously enhancing mass about 6 × 9.5 cm with central necrosis in the upper abdomen that appears to be originating from the gastric antrum (greater curve). The mass was highly suggestive of GIST based on CT; it was the same mass that was previously misdiagnosed as a liver hemangioma (Figure 1). Endoscopic ultrasound confirmed the previous findings. However, no biopsy was attempted due to the risk of bleeding.

**Figure 2:**
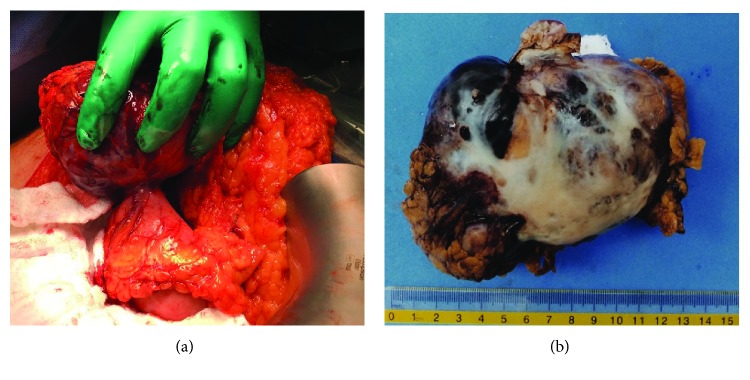
(a) Intraoperative photography of pedunculated gastric mass. (b) Gross examination of the mass, measures around 10 × 7 × 6 cm.

**Figure 3:**
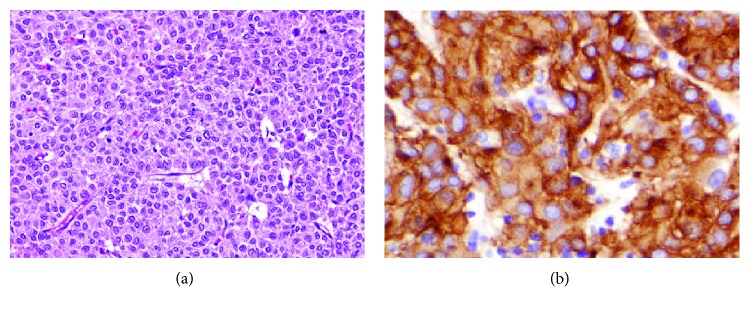
Microscopic examination of the gastric mass. (a) Epithelioid GIST of the stomach with rounded nuclei and a clear cytoplasm. (b) Epithelioid GIST strongly staining for DOG1.

Histopathological examination of the GEJ polyp revealed tubulovillous adenoma with elements of adenocarcinoma in situ. The patient was admitted with a provisional diagnosis of early-stage adenocarcinoma of GEJ along with the incidental finding of enlarging gastric GIST. A trial of endoscopic mucosal resection of GEJ polyp was attempted but failed because of the polyp location that created a technical difficulty. Therefore, the patient was taken to the operating room with a plan to perform a wedge resection of the gastric mass and a submucosal resection of GEJ polyp through the same gastric opening. We planned to use frozen section (FS) to document negative margin resection and determine the need for a formal esophagectomy. Intraoperatively; a large (10 × 7 × 6 cm), extraluminal pedunculated mass was found at the posterior wall of the greater curvature of the stomach (Figure 2). Wedge resection of the gastric mass with negative margins was achieved along with a transgastric submucosal resection of the GEJ polyp. Fortunately, the FS examination of the polyp showed negative margins as well with no evidence of deep invasion. Postoperatively, the patient had a smooth course and was discharged home in a stable condition. The final pathological examination revealed a GEJ polyp around 1.7 × 1.4 × 0.6 cm. Microscopically, there was a focus of invasive adenocarcinoma involving the superficial submucosa of the polypoid lesion, negative margins, and no lymphovascular invasion (T1a NxM0). Furthermore, the gastric wall mass measured around 10 × 7 × 6 cm with a 2 × 1.5 cm stalk. Histopathology revealed encapsulated high-grade epithelioid GIST tumor with negative margins (pT3). The mitotic rate of 6/50 HPF and immunohistochemical stains were positive for DOG1 and CD34 but negative for CD117 (c-Kit) (Figure 3).

The final diagnosis was synchronous early-stage GEJ adenocarcinoma and a high-grade gastric GIST. Therefore, the patient was started on adjuvant imatinib treatment, along with endoscopic surveillance every six months and proton pump inhibitors.

## 3. Discussion

The term GIST was first coined by Mazur and Clark [4]. Although they account for only 0.1–3% of all GI malignancies, GISTs are the most common mesenchymal tumors of the GI tract. They can arise anywhere along the GI tract with a preferred gastric location in about 60% of the cases, followed by the small bowel (25–35%), colon and rectum (5–10%), and esophagus (<5%). They originate from the intestinal cells of Cajal or their precursors [1, 2]. GISTs are composed of either spindle (70%) or epithelioid (20%) cells or mixed. They are usually positive for c-Kit (CD117), CD34, and DOG1 immunostaining [5]. DOG1 was discovered in 2004 and showed strong positivity in different histological types of GIST [6]. It is a highly sensitive and specific marker for detecting GISTs of gastric origin, those of epithelioid morphology and those harboring PDGFRA mutation [6, 7]. Lopes et al. found that DOG1 was positive in 87–94%, while CD117 and CD34 were positive in 74–95% and 60–70% of the examined GIST cases, respectively [8].

GISTs usually arise from the stomach wall and extend inward toward the mucosa or outward toward the serosa, while pedunculated GIST is a unique pattern of growth that has rarely been reported [9, 10]. Many GISTs are diagnosed after the onset of clinical symptoms including abdominal mass, pain, and bleeding [11]. However, occasionally they are discovered incidentally during the evaluation of other clinical entities [12]. GIST and other primary GI tract neoplasms are distinct tumors originating from different cell layers. Synchronous development of such carcinomas is uncommon [13]. The percentage of GIST with other diagnosed neoplasms has been reported to range between 3 and 33% [14]. Most cases involve adenocarcinomas, lymphomas, carcinoids, or leiomyosarcomas of the stomach [15].

Concurrent occurrence of GIST and GEJ neoplasms is rare; only a few cases have been reported in the literature [3]. Spinelli et al. reported a case with squamous cell carcinoma of the lower third of the esophagus with an incidental pathologic diagnosis of a concomitant GIST in the thoracic tract [11].

Similarly, Hsiao et al. reported a 75-year-old man who had a concurrent GIST and adenocarcinoma at the GEJ [3], in addition to a case series by Chan et al. who documented 4 cases with coexistence of GEJ adenocarcinomas and gastric GISTs [16].

Synchronous occurrence of gastric epithelial and stromal tumor raises the question whether such an occurrence is a simple coincidence or the two lesions are related to a certain etiology [17]. Various hypotheses have been proposed regarding this simultaneous presentation, including gene mutation such as c-Kit mutations, expression of metallothioneins, and *H. pylori* infection that may promote proliferation of different cell lines, while other authors considered the possibility of sporadic occurrence especially in countries that exhibit a high incidence of gastric cancer, such as Japan. Currently, there is no strong data to support such hypotheses [3, 5, 17].

Surgery has been so far the most effective treatment modality for GIST. Resection is usually accomplished with a wedge resection of the stomach, whereas formal gastric resection is occasionally required for larger and difficultly located GISTs [13]. High-grade GISTs, recurrent cases, metastatic diseases, and unresectable tumors can be treated with tyrosine-kinase inhibitors, such as imatinib [18]. While surgical resection remains the mainstay of treatment of resectable GIST, neoadjuvant imatinib may be preferred in a potentially resectable tumor if a reduction in tumor size would significantly decrease the morbidity of surgery. However, proof of the survival and effectiveness of neoadjuvant imatinib has not been sufficiently justified. Tumor biopsy should be performed to confirm the diagnosis and tumor genotype before establishing a neoadjuvant treatment [19].

Most of the GISTs that have been reported with simultaneous neoplasms were small with low mitotic count and very low risk of invasion [5, 14, 16]. Our case seems to be unique in that the GIST was the larger lesion and it was composed of a high-grade epithelioid component with a high mitotic rate. Although the GIST was large, wedge resection of the stomach was enough to achieve negative margins. This was due to its pedunculated nature. Transgastric submucosal resection of the GEJ polyp was accomplished through the same stomach incision, with negative surgical margins. In our case, the GEJ polyp could not be resected endoscopically due to technical difficulties. Laparoscopic surgery would be an option but we decided to proceed with an open surgery because of the large GIST tumor and the difficult position of the GEJ polyp. There was no role for neoadjuvant therapy as the tumor was resectable and no preoperative biopsy was performed. Since the GEJ polyp was an early-stage adenocarcinoma and the GIST a high-risk tumor, therefore the patient was started on adjuvant imatinib treatment along with endoscopic surveillance every six months and proton pump inhibitors.

## 4. Conclusion

We report a rare case of synchronous gastric GIST and a GEJ polypoid adenocarcinoma. Further studies are needed to analyze the correlation between the simultaneous occurrence of GISTs and other primary GI neoplasms.

## References

[1] Kaur R., Bhalla S., Nundy S., Jain S. (2013). Synchronous gastric gastrointestinal stromal tumor (GIST) and other primary neoplasms of gastrointestinal tract: report of two cases. *Annals of Gastroenterology*.

[2] Miettinen M., Lasota J. (2001). Gastrointestinal stromal tumors--definition, clinical, histological, immunohistochemical, and molecular genetic features and differential diagnosis. *Virchows Archiv*.

[3] Hsiao H. H., Yang S. F., Liu Y. C., Yang M. J., Lin S. F. (2009). Synchronous gastrointestinal stromal tumor and adenocarcinoma at the gastroesophageal junction. *The Kaohsiung Journal of Medical Sciences*.

[4] Mazur M. T., Clark H. B. (1983). Gastric stromal tumors. Reappraisal of histogenesis. *The American Journal of Surgical Pathology*.

[5] Cai R., Ren G., Wang D. B. (2013). Synchronous adenocarcinoma and gastrointestinal stromal tumors in the stomach. *World Journal of Gastroenterology*.

[6] Swalchick W., Shamekh R., Bui M. M. (2015). Is DOG1 immunoreactivity specific to gastrointestinal stromal tumor?. *Cancer Control*.

[7] Guler B., Ozyilmaz F., Tokuc B., Can N., Tastekin E. (2015). Histopathological features of gastrointestinal stromal tumors and the contribution of DOG1 expression to the diagnosis. *Balkan Medical Journal*.

[8] Lopes L. F., West R. B., Bacchi L. M., van de Rijn M., Bacchi C. E. (2010). DOG1 for the diagnosis of gastrointestinal stromal tumor (GIST): comparison between 2 different antibodies. *Applied Immunohistochemistry & Molecular Morphology*.

[9] Cavallaro G., Sadighi A., Polistena A. (2008). Pedunculated giant GISTs of the stomach with exophytic growth: report of two cases. *International Journal of Surgery*.

[10] Pugh J. L., Jie T., Bhattacharyya A. K. (2013). Pedunculated gastrointestinal stromal tumor (GIST) of the stomach presenting as pancreatic mucinous cystadenocarcinoma: a case report. *Journal of Clinical & Experimental Pathology*.

[11] Spinelli G. P., Miele E., Tomao F. (2008). The synchronous occurrence of squamous cell carcinoma and gastrointestinal stromal tumor (GIST) at esophageal site. *World Journal of Surgical Oncology*.

[12] Ulusan S., Koc Z., Kayaselcuk F. (2008). Gastrointestinal stromal tumours: CT findings. *The British Journal of Radiology*.

[13] Zhou Y., Wu X. D., Shi Q., Jia J. (2013). Coexistence of gastrointestinal stromal tumor, esophageal and gastric cardia carcinomas. *World Journal of Gastroenterology*.

[14] Liszka L., Zielińska-Pająk E., Pająk J., Gołka D., Huszno J. (2007). Coexistence of gastrointestinal stromal tumors with other neoplasms. *Journal of Gastroenterology*.

[15] Nakamura S., Aoyagi K., Iwanaga S., Yao T., Tsuneyoshi M., Fujishima M. (1997). Synchronous and metachronous primary gastric lymphoma and adenocarcinoma: a clinicopathological study of 12 patients. *Cancer*.

[16] Chan C. H. F., Cools-Lartigue J., Marcus V. A., Feldman L. S., Ferri L. E. (2012). The impact of incidental gastrointestinal stromal tumours on patients undergoing resection of upper gastrointestinal neoplasms. *Canadian Journal of Surgery*.

[17] Maiorana A., Fante R., Maria Cesinaro A., Adriana Fano R. (2000). Synchronous occurrence of epithelial and stromal tumors in the stomach: a report of 6 cases. *Archives of Pathology & Laboratory Medicine*.

[18] Chaudhry U. I., DeMatteo R. P. (2011). Advances in the surgical management of gastrointestinal stromal tumor. *Advances in Surgery*.

[19] Ishikawa T., Kanda T., Kameyama H., Wakai T. (2018). Neoadjuvant therapy for gastrointestinal stromal tumor. *Translational gastroenterology and hepatology*.

